# An efficient analog Hamming distance comparator realized with a unipolar memristor array: a showcase of physical computing

**DOI:** 10.1038/srep40135

**Published:** 2017-01-05

**Authors:** Ning Ge, Jung Ho Yoon, Miao Hu, E. J. Merced-Grafals, Noraica Davila, John Paul Strachan, Zhiyong Li, Helen Holder, Qiangfei Xia, R. Stanley Williams, Xing Zhou, J. Joshua Yang

**Affiliations:** 1 HP Labs, HP Inc., Palo Alto, California 94304, USA; 2 School of Electrical & Electronic Engineering, Nanyang Technological University, Nanyang Avenue, 639798, Singapore; 3 Department of Electrical and Computer Engineering, University of Massachusetts, Amherst, MA 01003, USA; 4 Hewlett Packard Labs, Palo Alto, California 94304, USA

## Abstract

We propose and demonstrate a novel physical computing paradigm based on an engineered unipolar memristor that exhibits symmetric SET switching with respect to voltage polarity. A one-dimensional array of these devices was sufficient to demonstrate an efficient Hamming distance comparator for two strings of analog states represented by voltages from the physical world. The comparator first simultaneously applies the two sets of voltages to the array of memristors, each of which is initially in its high resistance state and switches to its low resistance state only if the two voltages applied on that memristor differ by more than the switching threshold. An accurate analog representation of the Hamming distance is then obtained by applying a reading voltage to the memristors and summing all the resultant currents. The comparator with a small footprint can directly process analog signals and store computation results without power, representing a promising application for analog computing based on memristor crossbar arrays.

Physical computing systems utilize physics laws (e.g., ohm’s law) to directly sense and respond to the analog world and most efficiently store and output the computing results^1^,^2^. Emerging devices, such as memristors, may be engineered to enable this attractive computing paradigm. An efficient analog Hamming distance comparator based on memristor arrays demonstrated in this study serves as a typical example of a physical computing system based on new devices. For any pair of strings or words of equal length, the Hamming distance is defined as the total number of positions where the symbols or characters of the pair are different from each other in the corresponding coordinate positions. In other words, the Hamming distance measures the minimum number of changes required to transform one string into the other. It is a widely used concept in information theory, with numerous applications in coding, error correction and cryptography^3^,^4^,^5^,^6^,^7^,^8^,^9^,^10^. One example of the use of the Hamming distance is an estimate of the transmitted signal quality through counting the number of flipped bits in a fixed-length binary word. The Hamming distance is also used in genetic mapping by the counting number of nucleotide differences between two gene sequences^11^.

Any strings or words can be converted into a sequence code based on binary bits. The task of a Hamming distance comparator in a digital system is to count the number of binary bit flips that occur in the corresponding digits of two sequences. Using two 8-bit-streams as an example, 01101001 and 10101000 have a Hamming distance of three, i.e., there are flips in the first, second, and eighth bits. Implementing the comparator logic in hardware usually requires an array of 1-bit cells. Traditionally, the Hamming distance is calculated in a digital circuit, for example, by Exclusive OR (XOR) gates performing a bit-wise comparison of the incoming bits followed by a corresponding decision logic. A full sequence-length comparator circuit can be significantly faster, but at the expense of a larger circuit area and more logic gates. Sequence processing with fewer logic gates is more efficient in circuit area but requires more clock cycles. For digital circuits, the power consumption is proportional to the circuit capacitance and the number of signal transitions, making the efficiency of the traditional Complementary Metal–Oxide–Semiconductor (CMOS) approach low. This is greatly exacerbated when the word or string comparison involves an analog input signal, which requires an analog-to-digital converter (ADC). In this paper, we present a simple yet efficient architecture to perform analog Hamming distance computation utilizing memristors, which are two-terminal passive devices that change resistance upon electrical stimulation and stay in a non-volatile state after the switching. Memristors have attracted significant attention as a potential next generation non-volatile memory^12^,^13^,^14^,^15^,^16^,^17^,^18^,^19^,^20^,^21^,^22^. Efforts have also been made recently to utilize these devices for data processing in new applications such as analog and neuromorphic computing^23^,^24^,^25^,^26^,^27^,^28^,^29^,^30^,^31^,^32^, pattern recognition^33^,^34^,^35^, security applications^36^,^37^ and so on. In one embodiment of these devices, the resistance can be electrically switched between two states: a high-resistance state (HRS) and a low-resistance state (LRS). The switching from the HRS to LRS is called “SET” or “ON” switching, and conversely from the LRS to HRS is called “RESET” or “OFF” switching. Nonvolatile memristors can be classified into two categories: bipolar and unipolar (or nonpolar). Bipolar switching refers to the case where one voltage polarity is used for SET while the opposite polarity is required for RESET. In contrast, the SET and RESET in unipolar switching can occur with the same polarity of the applied voltage^38^,^39^. Recently, in the newly developed diffusive memristors^32^, the OFF/ON ratio could be over 1E10. In addition, these diffusive memristors can relax back to the HRS under zero bias, which negates the process of resetting all the devices and can be favorable in some applications.

In this study, we designed unipolar memristors with an electrically symmetrical SET-switching and a large ON/OFF conductance ratio, as presented in Fig. 1. Figure 1(a) shows an optical microscopic image of a 32 × 32 memristor crossbar (MC) and the inset to Fig. 1(a) schematically illustrates the device structure. TiO_2_-based unipolar memristors were fabricated with symmetrical Pd electrodes. A thin Ta layer was deposited in order to enhance the adhesion of the bottom electrode to the SiO_2_/Si substrate. In addition, an Au layer was inserted between the Pd and the Ta layers in the bottom electrode and also deposited on the Pd layer in the top electrode to reduce the line resistance of the 32 × 32 MC. Figure 1(b) shows SET and RESET switching operations, which can be obtained with either positive or negative voltages (bottom electrode was grounded) with good symmetry and reproducibility in both voltage and current. In addition, by optimizing the thickness and fabrication conditions, an electroforming process was not needed to precondition the device for the subsequent SET and RESET switching operations. The SET was performed by using a quasi-DC voltage sweep with a current compliance, while no current compliance was used for RESET. The SET and RESET symmetries are important for a Hamming distance comparator because the voltage difference could be positive or negative depending on the amplitudes of the reference and input voltages. Figure 1(b) also shows the device has an ON/OFF conductance ratio of ~1000 at 0.2 V. The large ON/OFF conductance ratio increases the signal to noise ratio (SNR) by suppressing the background OFF current and enables a larger MC size for longer string comparison. Figure 1(c) and (d) present the measured variances of the operation voltages from cycle to cycle and from device to device, respectively. The average and standard deviation for the ON states of both polarities was 1.52905 ± 0.20446 V for positive and −1.59078 ± 0.19791 V for negative polarity. The uniform distributions of the SET and RESET threshold voltages demonstrated in the fabricated devices determined the accuracy of the Hamming distance comparator.

For digital circuits, the voltage level for the bit “1” or “0” is usually assigned a fixed value. For example, 3 V may be assigned to represent bit “1” and 0 V to represent bit “0”. There will always be DC voltage variations and AC fluctuations for analog signals. For our Hamming distance comparator, it is extremely valuable to perform direct analog comparisons without going through an ADC. This is important for applications in the internet of things (IoT) and wearable devices, since data gathered by sensors are usually in analog form and a Hamming distance needs to be computed against a known reference. For example, the outputs from temperature sensors and motion sensors are analog voltage levels within a predetermined range. If we compute the Hamming distance using the sum of currents via Kirchoff’s Laws, we want the total current to be linearly proportional to the number of different bits. In other words, we need the unipolar memristors to be always SET to the same level of resistance with input voltages of varying amplitudes (corresponding to different comparison codes), but over any SET switching threshold voltage. Two-wire electrical characterizations were carried out by applying defined voltages across the unipolar devices. Figure 2 shows the input switching voltage conditions and post switching current readings on the corresponding devices. As seen in Fig. 2(a), the 32 input voltages from 0 V to 5 V were applied to one electrode of each of the 32 diagonal devices, while the other electrode was grounded. A compliance current of 1 mA was enforced during the SET switching to limit over-switching and prevent permanent breakdown of a memristor. One of the key factors is to produce the same LRS in all the devices, which requires that the conduction channels in all the switched memristors to have similar sizes. Figure 2(b) shows the measured currents for all the memristors pre- and post-switching using a 0.2 V reading voltage. For any applied voltage amplitude below 1.5 V, a device remained in the HRS with a resistance of around 0.2 Mohm. Devices were switched to a LRS around 400 ohm for >2 V voltage amplitude difference (referenced from Ground). With a current compliance, the unipolar memristors exhibited nearly identical LRS and HRS after SET or RESET switching, respectively. This provides the required linear correlation between the summed current and the number of devices switched in the comparator circuit.

Using the unipolar memristor discussed above, a fully populated MC (i.e. a switch device in every row and column junction) was constructed for the demonstration of Fig. 3. Just a diagonal array of the devices was utilized, since only N diagonal memristors are needed for an N-bit comparison for this Hamming distance computation. More compact circuit geometries are possible in practical applications as shown in Fig. 3(d,e), but the experiments and measurements here took advantage of existing memristor crossbar fabrication procedures and electrical measurement facilities. The compact circuit will enjoy better accuracy since there will be essentially no sneak path current. There are three primary steps to compute the Hamming distance for N bit strings (A_n_…A_2_A_1_) and (B_n_…B_2_B_1_), as shown in Fig. 3(b):

Step 1: All unipolar memristors are RESET to the HRS. This ensures the Hamming distance comparator operation is not affected by previous operations.

Step 2: The bit strings (A_n_…A_2_A_1_ and B_n_…B_2_B_1_) are input using their corresponding voltage levels (digital or analog values) to the rows and columns, respectively, as shown in Fig. 3(a). If bits A_i_ and B_i_ have the same voltage or the voltage difference between them is less than the threshold, the memristor at the cross-point of the i^th^ Row and i^th^ column will remain in the HRS. However, if bits A_i_ and B_i_ have a voltage difference greater than the threshold, the unipolar memristor will switch into the LRS state with the compliance current enforced on either the rows or columns.

Step 3: A small read voltage is subsequently applied to the columns (or rows) of the crossbar and the rows (or columns) are grounded. The summed current from the MC, which is an analog value for the Hamming distance, is measured and can be translated to a digital value using a trans-impedance amplifier (TIA) and an experimentally calibrated ADC.

Figure 4 shows two examples of analog string comparison. Figure 4(a)–(c) are case 1 with a Hamming distance of 15: Fig. 4(a) presents the actual voltages for the two strings to be compared; Fig. 4(b) shows the voltages applied on each device normalized by the corresponding reference voltage, which can be used to easily determine whether a device will be switched or not; Fig. 4(c) shows the pre-comparison validation (all devices in the HRS, black squares) and the post comparison devices (red dots) with high currents for those devices that switched to their LRS states. In this case, 15 devices were switched, yielding a Hamming distance of 15, which corresponds to a measured total current of 7.97 mA. Figure 4(d)–(f) provide another example with a Hamming distance of 13, corresponding to a current of 6.94 mA. Figure 5 shows a plot of the total measured current vs. the number of switched devices, which exhibits a fairly linear relationship. The current contribution from the unswitched devices is negligible because of the very large ON/OFF conductance ratio of the memristors. As an example, when the measured total current was 7.97 mA, the estimated number of switched memristors from the linear fit was 15.36 and the actual Hamming distance was 15. Thus, the Hamming distance comparator demonstrated reasonable accuracy even in the case of analog value comparison.

In conclusion, we have constructed and demonstrated a new physical computing system, which directly senses analog inputs and performs computing within one step. The output results are stored in the computing system without requiring energy. Specially designed unipolar memristors are utilized for this demonstration. The results here provide an encouraging example and a pathway toward physical computing using emerging electronic devices.

## Experimental Procedure

### Pd/TiO_2_/Pd memristor device fabrication

Standard photolithography and lift-off processes for top and bottom Pd electrodes were used for the MC fabrication. The device area of an individual memristor was 5 μm × 5 μm. In an sputtering system (AJA international, Orion 8), around 20-nm-thick TiO_2_ layer was deposited on a 30-nm-thick Pd/Au(50 nm)/Ta(3 nm)/SiO_2_/Si substrate using a commercial TiO_2_ target under a 7 mtorr of Ar (20 sccm) and O_2_ (5 sccm) ambient. The Pd bottom electrode, Au and Ta layers were deposited by thermal evaporation system (CHA, SE-600). Finally, a 30-nm-thick Pd top electrode and then a 50-nm-thick Au were deposited by thermal evaporation system.

### Electrical Measurements

DC electrical characterizations and MC switching operations were carried out using an Agilent B1500A Precision Semiconductor Parameter Analyzer.

## Additional Information

**How to cite this article**: Ge, N. *et al*. An efficient analog Hamming distance comparator realized with a unipolar memristor array: a showcase of physical computing. *Sci. Rep.* **7**, 40135; doi: 10.1038/srep40135 (2017).

**Publisher's note:** Springer Nature remains neutral with regard to jurisdictional claims in published maps and institutional affiliations.

## Figures and Tables

**Figure 1:**
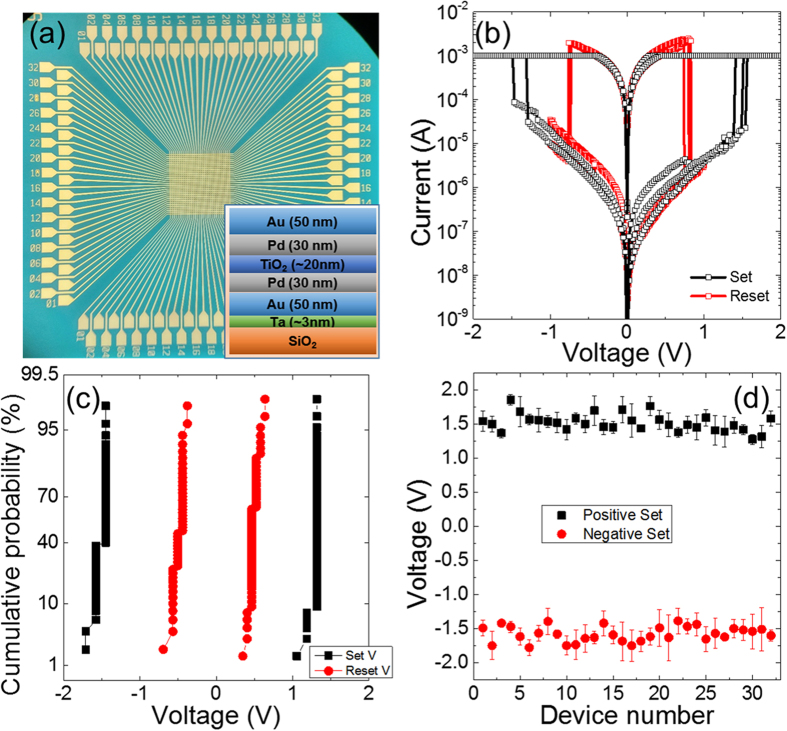
Purpose-designed unipolar memristor and crossbar for Hamming distance computation. (**a**) Optical microscopic image of a 32 × 32 memristor crossbar. The inset is a schematic illustration of the device stack structure. (**b**) Semi-log plot of the I–V curves for switching. **(c**) Cycle-to-cycle variability of a single device. (**d**) Device-to-device variability for 32 memristors. The average and standard deviation for the ON states of both polarities was 1.52905 ± 0.20446 V for positive and −1.59078 ± 0.19791 V for negative polarity.

**Figure 2:**
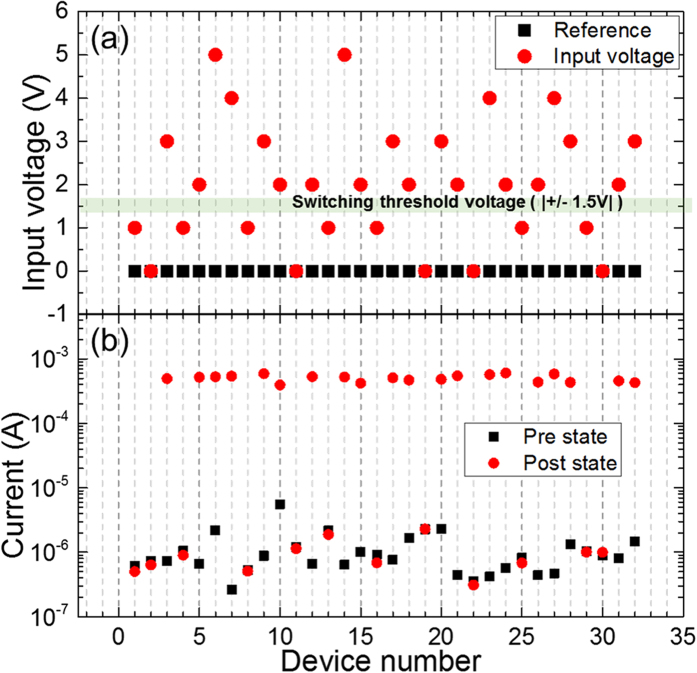
Input voltages and the corresponding pre- and post-switching currents for 32 memristors. (**a**) With one electrode grounded, input voltages ranging from 0 V to 5 V were applied to the other electrode of each of the 32 devices. A compliance current of 1 mA was enforced to control the resistance level of the LRS. (**b**) Measured current through each memristor pre- and post-switching using a 0.2 V reading voltage.

**Figure 3:**
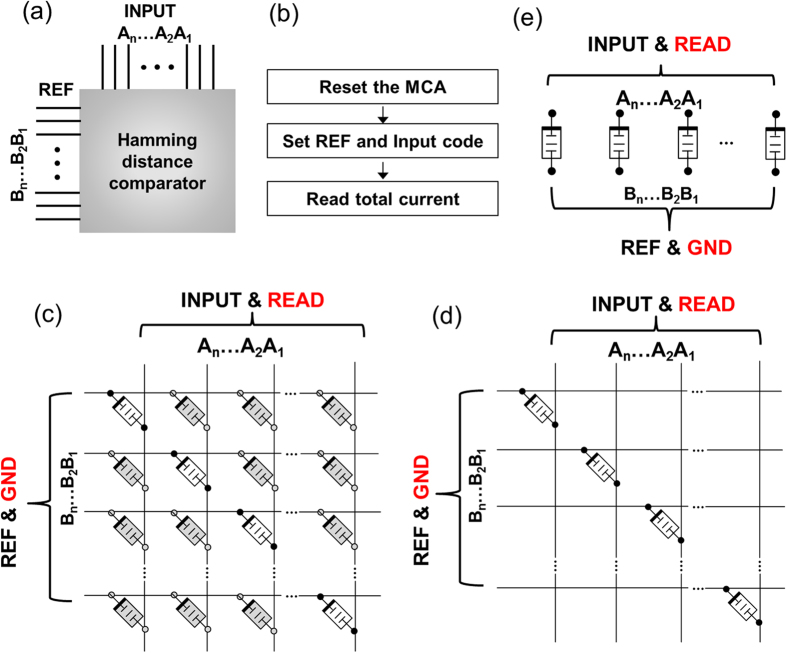
Hamming distance comparator. (**a**) Schematic illustration of the core circuit architecture. (**b**) Steps for the comparator operation. (**c**) Active unipolar memristors on a full-populated crossbar with only diagonal devices utilized. The input and read conditions are indicated. The rest of the memristor devices highlighted in grey color are un-used devices and they will remain in the HRS all the time, which will have minimum impact to the circuit operation. (**d**) Unipolar memristors only exist on the diagonal line of a crossbar with the input and read conditions indicated for practical application. (**d**) Unipolar memristors on the in-line architecture with the input and read conditions indicated for practical application.

**Figure 4:**
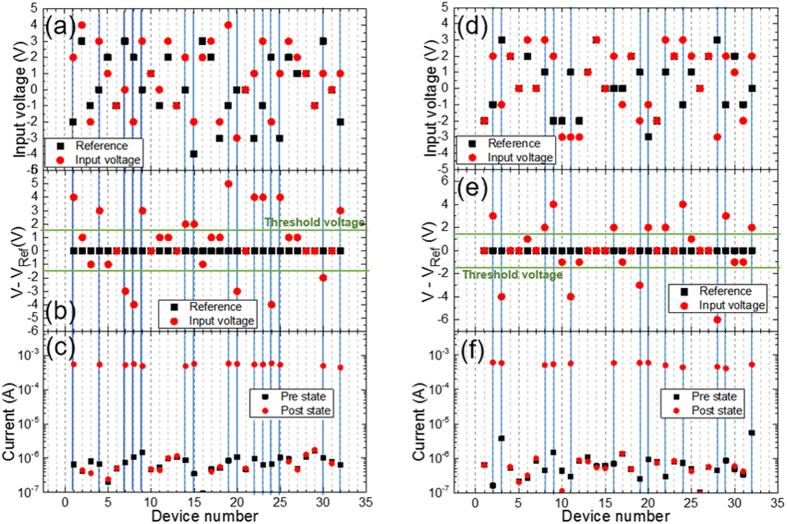
Two Hamming distance computations based on analog input values. (**a**–**c**) Case 1 with Hamming distance = 15. (**a**) The actual voltages for the two strings to be compared; (**b**) the normalized voltage differences by setting reference voltages to 0 for each device; (**c**) pre-comparison validation (all devices are in the HRS) and current readings of post-comparison devices, some of which exhibit the LRS with a high current level. In this case, 15 devices have been switched, corresponding to a Hamming distance of 15. (**d**–**f**) Similarly for case 2 with a Hamming distance equal to 13.

**Figure 5:**
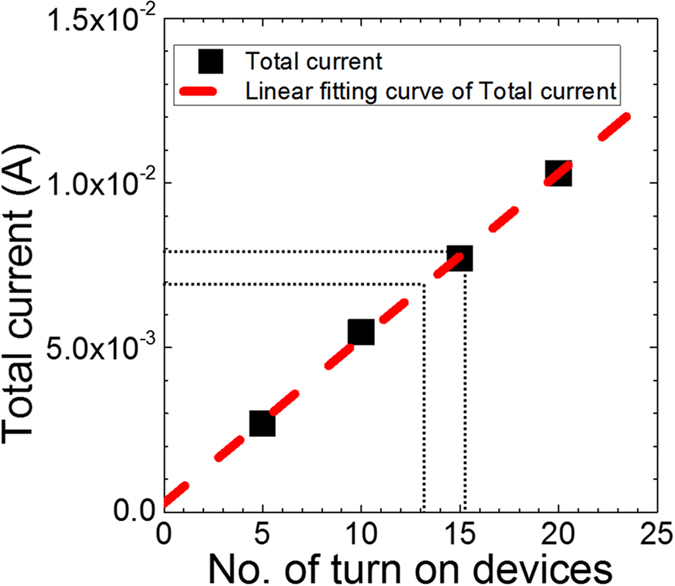
Total current vs. number of switched devices in the memristor crossbar. The resultant plot yields a linear fit of = 2.72 * 10^−4^***x*** + 5.01 * 10^−4^. The equation parameters may change with different unipolar devices used.
